# Association between sinus septa and lateral wall thickness with risk of perforation during maxillary sinus lift surgery: A systematic review and meta-analysis

**DOI:** 10.1371/journal.pone.0308166

**Published:** 2024-08-08

**Authors:** Baohua Yang, Tiantian Wang, Yuzhen Wen, Xingguang Liu

**Affiliations:** 1 Department of Stomatology, Zibo Central Hospital, Zibo City, Shandong Province, China; 2 Department of Oral and Maxillofacial Surgery, Zibo Central Hospital, Zibo City, Shandong Province, China; 3 Department of Oral and Maxillofacial Surgery, School and Hospital of Stomatology, Cheeloo College of Medicine, Shandong University, Jinan City, Shandong Province, China; Stomatology Hospital of Guangzhou Medical University, CHINA

## Abstract

**Objective:**

Sinus membrane perforation is a common complication of sinus lift surgery. This review aimed to examine if anatomical factors such as the presence of septa and lateral wall thickness influence the risk of membrane perforation.

**Methods:**

This study was registered on PROSPERO (CRD42023488259). PubMed, Embase, and Web of Science were searched for relevant studies published up to 26th June 2024. The outcome of interest was the risk of perforation based on presence of septa and lateral wall thickness. Random-effects meta-analysis was conducted with dichotomous data to obtain the odds ratio (OR) of perforation using Review Manager.

**Results:**

Ten studies with 1865 patients undergoing 2168 “lateral” sinus lift procedures were included. The total incidence of Schneiderian membrane perforations was 19% (405 cases). Schneiderian membrane perforation was present in 169/425 cases (39.76%) with sinus septa and 184/1492 cases (12.33%) without septa. Meta-analysis showed that septa were significantly associated with an increased risk of perforation (OR: 4.03 95% CI: 1.77, 9.19) with high heterogeneity (I^2^ = 87%). The certainty of the evidence was very low. Data on lateral wall thickness and risk of perforation was too heterogeneous for a meta-analysis. Studies reported mixed results on the risk of perforation based on lateral wall thickness.

**Conclusions:**

Our results show, with very low-quality evidence, that the presence of septa significantly increases the risk of perforations during maxillary sinus lift surgery. Evidence on the association between lateral wall thickness and a risk of perforations during sinus lift surgery is conflicting, and no clear conclusions can be derived at this stage.

## Introduction

Rehabilitation of posterior maxillary teeth with dental implants is often complicated by the maxilla’s porous nature and the maxillary sinus’s proximity. Pneumatization of the maxillary sinus with age frequently leaves minimal residual bone height for an adequate length of dental implants [1]. Management usually involves sinus augmentation procedures using either the lateral window- or the osteotome-mediated transalveolar technique [2]. According to the current guidelines, the lateral window technique should be used in cases of residual bone height less than 5mm. In comparison, the osteotome-mediated transalveolar technique is recommended for bone heights >5mm [3].

High implant success/survival rates up to 88–100% can be achieved when dental implants are placed in combination with a sinus augmentation procedure [4, 5]. However, sinus lift involves delicate elevation of the sinus membrane of the maxillary bone. Since the average thickness of the membrane is <1mm, it is prone to perforation during different stages of the procedure, such as the creation of the bony window through the lateral wall, lifting of the actual membrane, graft placement, or positioning of the implant [6]. Small perforations can either be sutured or closed by a collagen membrane. However, large perforations may require stopping the procedure and redoing the surgery at a later time [7]. Therefore, to minimize the risk of possible complications, the surgeon needs to be aware of the anatomical factors that can increase perforation rates during sinus lift surgery. Several features of the maxillary sinus have been recognized as risk factors for perforation, including maxillary contours, thin sinus membrane, septa, and lateral wall thickness [8, 9].

However, few meta-analyses have reviewed evidence on anatomical risk factors for sinus membrane perforations. Monje et al [10] have shown that thick sinus membranes are associated with a reduced risk of perforations as compared to thinner membranes. Fang et al [11] have shown that pathological sinus membranes do not correlate with a higher risk of perforations compared to normal sinus membranes. Moreover, the occurrence of sinus membrane thickening, and pseudocyst did not affect implant failure rates. To date, no systematic review has examined whether bony features such as the presence of septa and lateral wall thickness increase the risk of membrane perforations.

This systematic review and meta-analysis aimed to assess the impact of septa and thickness of the lateral wall of the maxillary sinus on the risk of sinus membrane perforation.

## Material and methods

### Focused question

The focused questions of the review were: 1) Does the presence of septa increase the risk of perforations in maxillary sinus lift surgery? 2) Is the thickness of the lateral wall a risk factor for perforations in lateral approach maxillary sinus lift surgery?

### Eligibility criteria

The following PICO-based criteria were used:

Population: Adult patients undergoing sinus lift surgery using the lateral window technique.

Intervention: Thick lateral wall or presence of septa.

Control: Thin lateral wall or absence of septa.

Outcome: Risk of perforation.

All types of studies published in English language were eligible.

Unpublished reports, non-peer-reviewed studies, theses, dissertations, editorials, and animal studies were excluded.

### Search strategy

A literature search and review were performed according to the PRISMA guidlines (S1 Table) [12]. Registration was done on PROSPERO (CRD42023488259). PubMed, Embase, CENTRAL, ScienceDirect, and Web of Science were searched by the two reviewers with the aid of a medical librarian from inception to 26th June 2024 using the following keywords: sinus membrane; Schneiderian membrane; sinus lift surgery; sinus augmentation surgery, lateral wall, septa, anatomy, complications, and perforation. The search strings are presented in S2 Table. The librarian and the study reviewers collated all search results and deduplicated them using the reference manager software EndNote X8 (Thompson ISI Research soft, Philadelphia, Pennsylvania). The remaining unique studies underwent preliminary screening by the two reviewers independently. Potentially relevant articles were sourced and further screened based on the inclusion criteria. References of studies were also screened to identify additional related articles and avoid missing data. Lastly, Google Scholar was scanned as a source of gray literature for additional studies. Any disagreements between reviewers were discussed and resolved.

### Data extraction and study quality

Information obtained from the studies included study authors, year, location, study type, sample size, age and sex details, type of sinus lift technique, method of drilling, number of cases with septa, number of cases with perforation, mean thickness of the lateral wall and outcome data based on presence of septa and lateral wall thickness. Details were collected by the two reviewers using a data collection form. Perforation was defined as any disruption of the continuity of the sinus membrane. Perforation rates were calculated based on number of sinuses and not per patient.

The Newcastle-Ottawa Quality Assessment Scale (NOS) was used to evaluate the quality and the risk of bias [13]. The scale assesses three main aspects of the study: selection (0–4 points), comparability (0–2 points), and outcomes (0–3 points), with higher scores indicating higher quality and lower risk of bias. Two reviewers independently assessed the risk of bias and disagreements were resolved by discussion.

### Statistical analysis

“Review Manager” (RevMan, version 5.3; Nordic Cochrane Centre (Cochrane Collaboration), Copenhagen, Denmark; 2014) was used for the analysis. Dichotomous data were pooled to generate odds ratio (OR) with 95% confidence intervals (CI). No data conversion was required for the meta-analysis. Funnel plots were examined for any publication bias. Heterogeneity between the studies was measured by the chi-square test and the I^2^ statistics that estimate what % of the variability in effect size is due to heterogeneity rather than sampling error. Any value >50% was considered substantial heterogeneity [14]. Sensitivity analysis was performed to evaluate the stability of results on sequential exclusion of studies. No subgroup or meta-regression was possible due to limited data. Certainty of evidence was examined using Grading of Recommendations Assessment, Development, and Evaluation (GRADE) criteria.

## Results

### Search results

**Fig 1:**
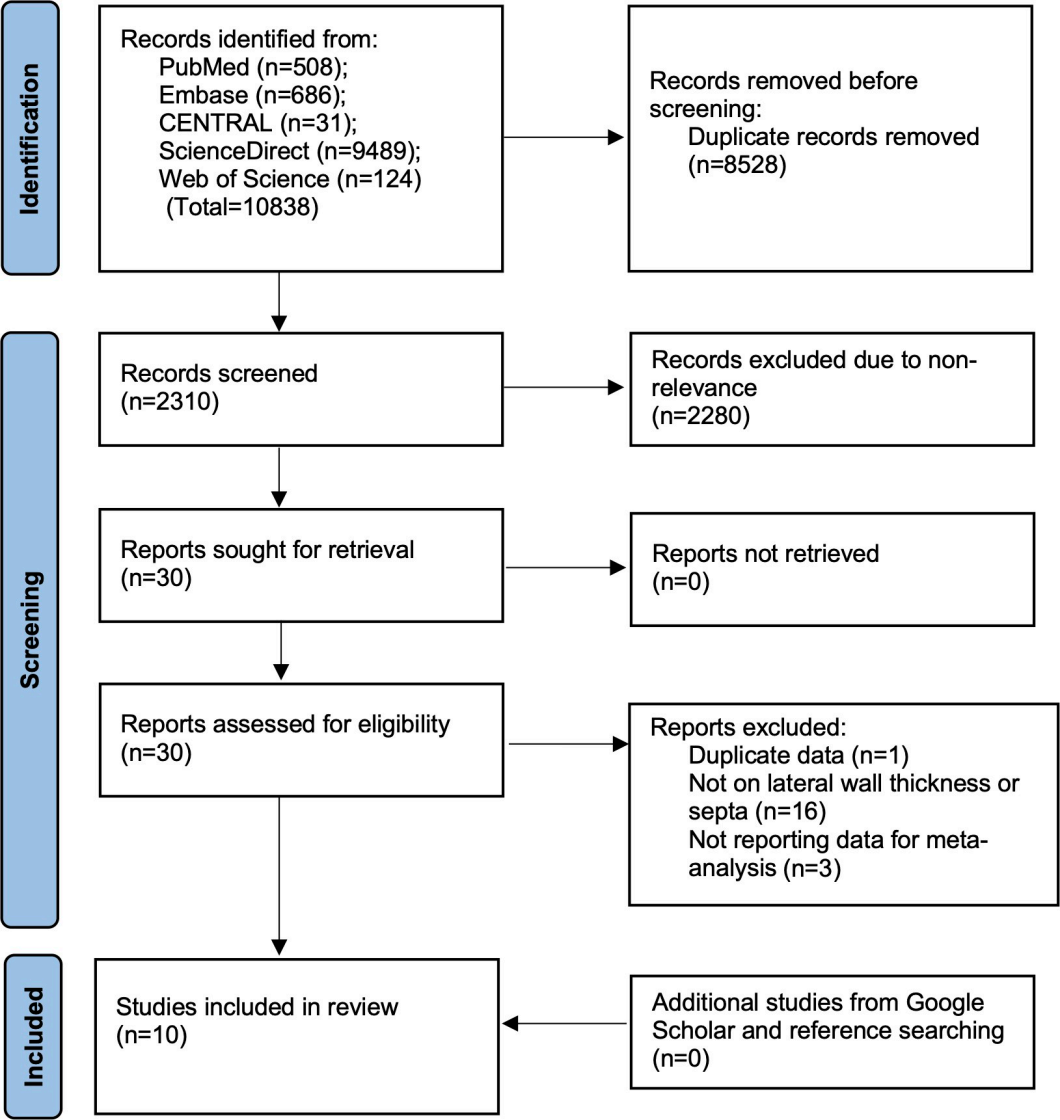
Study flow chart.

A systematic search of all databases resulted in the retrieval of 10838 articles. Of them, 8528 studies were removed as duplicates. Out of 2310 articles, 2280 were eliminated based on the initial screening, 30 studies underwent full-text analysis and ten were selected for the meta-analysis [15–24] (Fig 1). The list of excluded studies with reasons is presented in S3 Table. No additional studies were retrieved from Google Scholar or references of included studies. There were no inter-reviewer disagreements in the selection of studies (kappa = 1).

### Baseline details

The included observational studies were published between 2014 to 2023 and were from Switzerland, Austria, Turkey, China, Iran, and the USA (Table 1). Only one study [24] was prospective, while the rest were retrospective [15–23]. The combined sample size of all studies was 1865 patients with 905 males (48.5%) and 960 females (51.5%). Sample sizes of individual studies ranged from 77 to 355 patients. The total number of sinus lift procedures conducted was 2168. The number of sinus lift surgeries in the studies ranged from 73 to 434. Five studies [15, 17, 18, 21, 22] used both rotary and piezoelectric devices for the creation of the lateral window. Two studies [16, 20] did not report on the technique used for lateral window creation. The mean age of the patients varied from 50.3 to 64.4 years. The number of sinuses with septa ranged from 12 to 27% in the included studies. Overall, 455 of 2168 (21%) sinuses reported the presence of septa. Of 2168 sinus lift procedures, 405 resulted in sinus membrane perforation (19%). The quality of the included studies was moderate, with an average NOS score of NOS 7. Two points were deducted for each study as they did not control for the confounding factors between the study and the control groups. The inter-reviewer agreement for the risk of bias analysis was high (kappa = 0.95).

**Table 1:** Summary of the included studies.

Study	Location	Type	Sample size	Sinus lift procedures	Mean age (years)	Male patients	Method for drilling	Presence of septa	Lateral wall thickness (mm)	Number with perforation	Anatomical risk factors assessed	NOS score
Prajapati 2023 [17]	USA	Retrospective	99	122*	63.8	52	Rotary & piezoelectric	19	NR	17	Septa, SMT, pathologies	7
Nemati 2023 [24]	Iran	Prospective	140	140	54.6	81	Rotary	24	1.54± 0.97	22	Septa, mucous retention cyst, extent of edentulous area, lateral wall thickness, RRH, SMT	7
Shao 2021 [21]	China	Retrospective	278	278	50.3	148	Rotary & piezoelectric	34	NR	47	Septa, lateral wall thickness, sinus contours, RRH, SMT, blood vessels	7
Pizzini 2021 [16]	USA	Retrospective	166	202	64.4	102	NR	42	1.6± 0.6	52	Lateral wall thickness, window surface area, Angle formed between lateral and medial walls of sinus, Angle formed between lateral and medial walls of sinus	7
Krennmair 2021 [18]	Austria	Retrospective	355	434	55.9	174	Rotary & piezoelectric	125	NR	103	Septa, RRH	7
Basma 2021 [15]	USA	Retrospective	209	251	63.8	90	Rotary & piezoelectric	41	NR	67	Septa, Lateral wall thickness, RRH, residual ridge width, irregular sinus wall and floor, number of missing teeth	7
Tukel 2018 [19]	Turkey	Retrospective	120	120	53.5	70	Rotary	23	NR	22	Septa, RRH, mucous retention cyst	7
Marin 2018 [23]	Austria	Retrospective	121	137	55.1	52	Rotary	27	NR	19	Septa, lateral wall thickness, SMT, intraosseous blood vessels	7
Schwarz 2015 [20]	Austria	Retrospective	300	407	55.9	107	NR	110	NR	35	Septa, RRH	7
Arx 2014 [22]	Switzerland	Retrospective	77	77	57	29	Rotary & piezoelectric	20	NR	21	Septa, RRH, lateral wall thickness, SMT, membrane status, width of antrum	7

Abbreviation: NR, not reported; NOS, Newcastle Ottawa Scale; RRH, residual ridge height; SMT, sinus membrane thickness

*Data on septa was available only from 73 sinuses

### Association between the presence of septa and risk of membrane perforation

**Fig 2:**
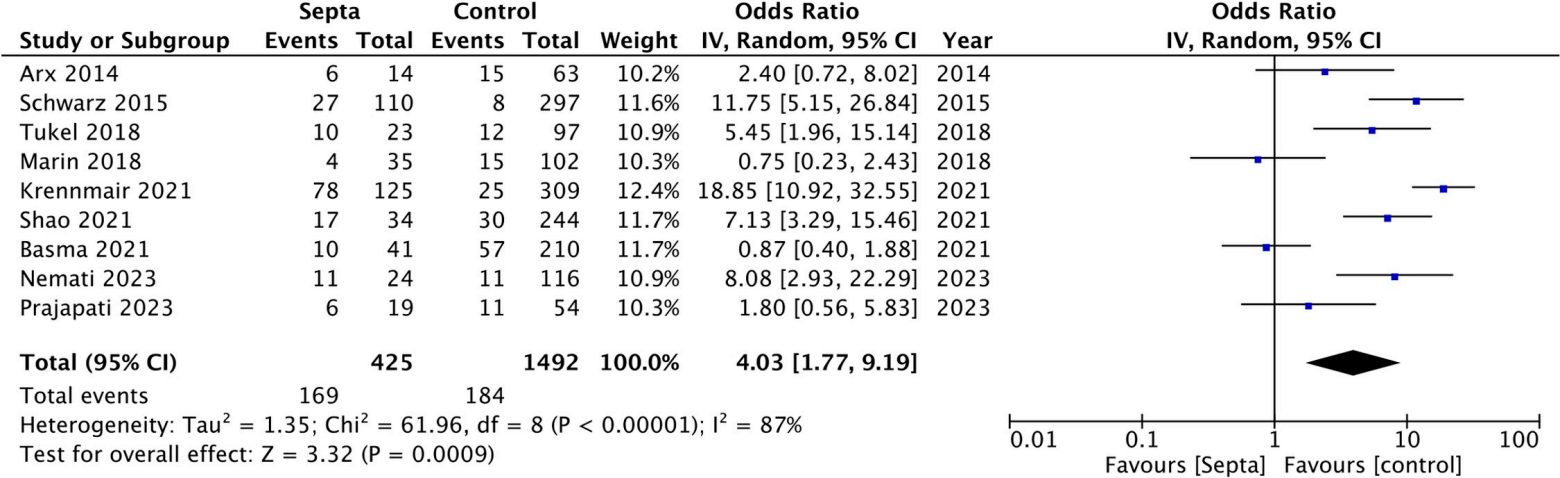
Meta-analysis of perforation rates based on the presence of septa.

Nine studies [15, 17–24] reported data on the risk of perforation based on the presence or absence of septa. Meta-analysis was conducted based on the perforation rates per number of sinuses and not per patient. The meta-analysis showed that the presence of septa was significantly associated with an increased risk of perforation (OR: 4.03 95% CI: 1.77, 9.19) (Fig 2).

**Fig 3:**
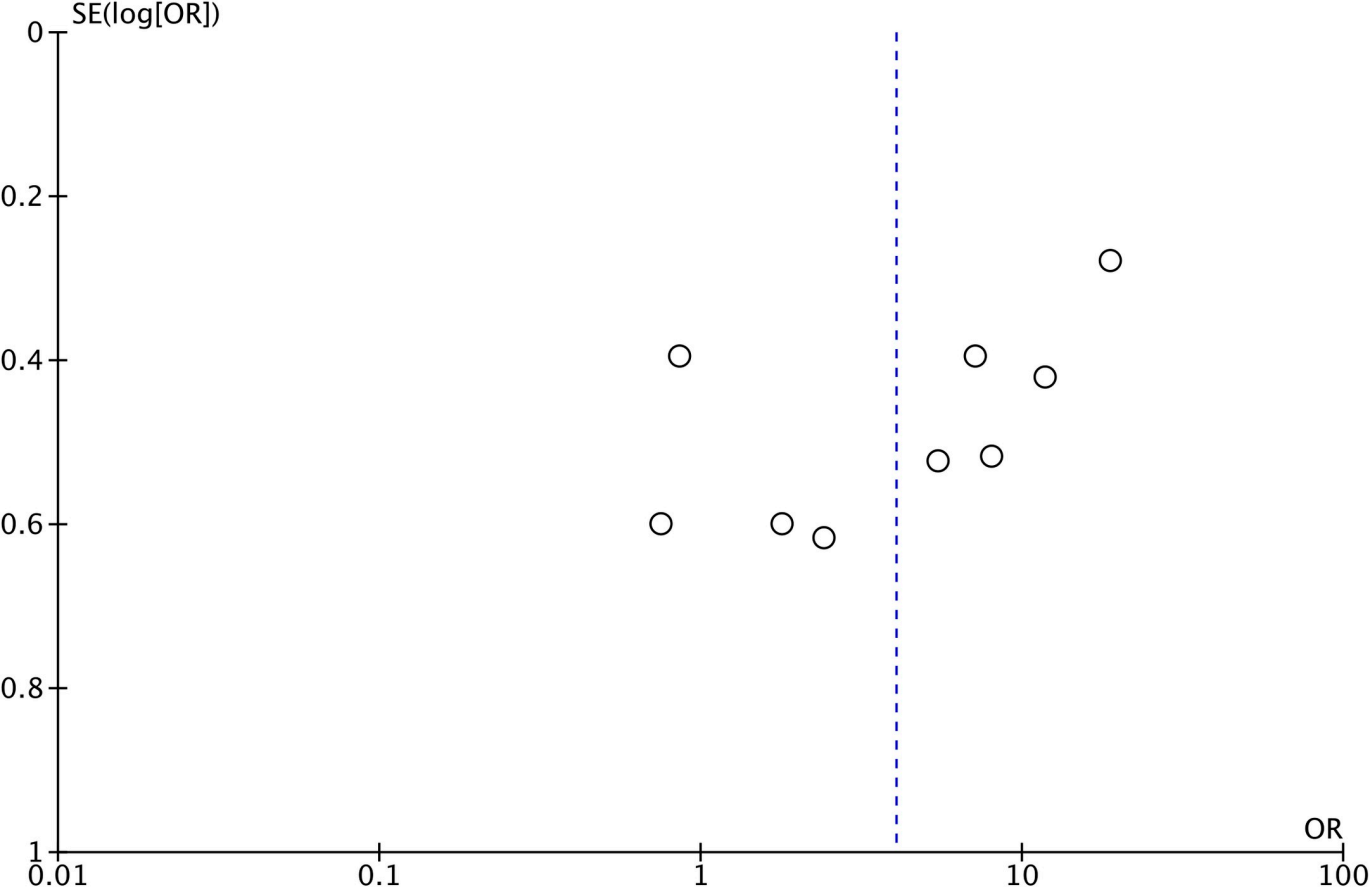
Funnel plot to examine publication bias.

There was high heterogeneity in the meta-analysis (I^2^ = 87%). The funnel plot did not show any major asymmetry indicating no evidence of publication bias (Fig 3).

A sensitivity analysis (Table 2) showed that the risk of Schneiderian membrane perforation in the group of patients with sinus septa was highest on the exclusion of the study of Basma et al [12], with OR of 5.07 and 95% CI: 2.53, 10.56 (I^2^ = 81%, p<0.001). On the exclusion of the study of Krennmair et al [15], the risk was lowest with an OR of 3.26 and 95% CI: 1.51, 7.04 (I^2^ = 80%, p = 0.003). However, the results remained significant on the exclusion of every single study suggesting that there was no outliner study in the meta-analysis. GRADE assessment of the evidence is shown in S4 Table. The overall certainty of the evidence was “very low”.

**Table 2:** Sensitivity analysis for the meta-analysis on the association of septa and sinus membrane perforation.

Excluded study	Odds ratio [95% Confidence intervals]	I^2^	P value
Prajapati 2023 [17]	4.42 [1.83, 10.65]	88	0.0009
Nemati 2023 [24]	3.69 [1.47, 9.22]	89	0.005
Shao 2021 [21]	3.71 [1.43, 9.61]	89	0.007
Krennmair 2021 [18]	3.26 [1.51, 7.04]	80	0.003
Basma 2021 [15]	5.07 [2.43, 10.56]	81	<0.001
Tukel 2018 [19]	3.87 [1.54, 9.70]	89	0.004
Marin 2018 [23]	4.91 [2.17, 11.12]	86	0.0001
Schwarz 2015 [20]	3.49 [1.39, 8.74]	88	0.008
Arx 2014 [22]	4.27 [1.75, 10.40]	88	0.001

### Association between lateral wall thickness and risk of membrane perforation

Data on the lateral wall thickness and a risk of perforation were too heterogeneous for a meta-analysis. Therefore, only a qualitative synthesis was conducted. Nemati et al [24] reported a statistically significant increased risk of perforation with each millimeter increase in lateral wall thickness (OR: 13.40 95% CI: 4.44–40.46). The mean lateral wall thickness in the perforation group was 2.95± 0.21mm while that of the non-perforation group was 1.28± 0.81mm. Pizzini et al [16] segregated their sample into three groups based on lateral wall thickness (<1.5mm, 1.5-2mm, and >2mm). Perforation rates were significantly lower in the <1.5mm group (14%) as compared to 1.5-2mm (39.3%) and >2mm groups (34.1%). Basma et al [15] also reported significantly thicker lateral walls in the perforation group vs the non-perforation group (2.43± 0.56 vs 1.21± 0.40). Perforation rates were 54.6% for thickness ≥2mm and 12.1% if the thickness was ≤1mm. Marin et al [23] reported no statistically significant difference in the risk of perforations based on the lateral wall thickness, with perforation rates of 10%, 19.3%, and 0% in cases with a lateral wall thickness of 0-1mm, 1-2mm, and >2mm, respectively. Similarly, Arx et al [22] noted no significant difference in perforation rates in patients with lateral wall thickness >1mm (28.3%) and ≤1mm (25.8%).

## Discussion

The iatrogenic perforation of the membrane is considered one of the most common complications during maxillary sinus lift surgery, with an incidence of about 11 to 56% [18, 25]. Any size of perforation of the lining is concerning as it causes a contact between the external environment and the graft material, and may alter the physiological function of the sinus, and increase the risk of sinusitis [26]. Another important concern is the survival of the implant itself. However, there is still no consensus on the impact of membrane perforation on implant survival. A case-control study by Park et al [27] that included 379 implants with a follow-up of 112 months have shown that sinus membrane perforation is not a risk factor for implant survival. A systematic review by Ragucci et al [28] included eight studies and showed that the survival rate of implants protruding in the maxillary sinus is about 95.6% and is independent of the level of penetration. Conversely, other studies have shown that implant survival is reduced in the case of sinus membrane perforations [29, 30]. A study by Lee et al [31] reviewed 85 reports and noted that each 10% increase in the prevalence of membrane perforations was associated with a 55% increase in the risk of reduced implant survival. Given the high incidence of sinus membrane perforations and their impact on the treatment, it is crucial to identify potential risk factors that can aid clinicians in planning the procedure and avoiding complications.

Maxillary sinus septa were first reported by Underwood in 1910 [32]. These are cortical plates of bone that divide the sinus into multiple compartments and could be in any region. According to a systematic review [33], about 28% of all maxillary sinuses have septa (ranging from 24 to 33%). Septa are identified in the premolar, molar, and retromolar regions with an incidence of about 24.4%, 54.6%, and 21.0%, respectively. In 87.6% of all cases, the direction of septa is transverse, while 11.1% of cases have sagittal, and 1.3% ‐ horizontal septa. Another recent meta-analysis by Henriques et al [34] has shown that the overall prevalence of septa per sinus is 33.2%, ranging from 27.8 to 38.5%, while the prevalence per patient ranges from 36 to 46%, with the mean prevalence of 41%. Septa are most commonly located in the middle area of the sinus and are transverse in direction (86%).

In general, all septa can be classified into two types: primary septa which are present since birth, and secondary septa which occur due to irregular pneumatization of the maxillary sinus. Given the variability in the number, size, location, and orientation of the septa, Wen et al [35] have proposed a classification system based on their clinical relevance. Maxillary sinus septa are classified as easy, moderate, and difficult based on their location as anterior to the zygomatic process, posterior to the zygomatic process, and both anterior and posterior to the zygomatic process, respectively. Easy and moderate types are always mediolateral in direction and singular in number in number, while the difficult type of septa are either singular and anteroposterior in direction or multiple and mediolateral. All three types are further subdivided based on the size (≤6mm or >6mm). Another classification of maxillary sinus septa that was proposed by Irinakis et al [36] is based on the direction of the septa and the chances of membrane perforation during sinus lift surgery. According to this classification, septa are divided into Class I, II, and III based on their orientation as mediolateral, anteroposterior, and horizontal, respectively. Chances of perforation increase from Class I to Class III subtypes. Furthermore, this classification method proposes an additional Class IV subtype, a combination of Classes I to III in orientation. Class IV septa is considered the most difficult and is associated with the highest risk of sinus membrane perforation [36]. While these classification systems recognize the role of septa as a risk factor for sinus membrane perforation, no systematic review has attempted to quantify the association of the risk of membrane perforation with the presence of sinus septa.

Our results showed that sinus septa were associated with a significantly increased risk of sinus membrane perforations. The risk of perforation was 39.8% in sinuses with septa and just 12.3% in sinuses without septa. A pooled analysis showed that the presence of septa increased the risk of perforation by about four times. The evidence was robust, as indicated by the sensitivity analysis, and the exclusion of individual studies failed to change the significance of the results. Nevertheless, heterogeneity remained high. The credibility of our review findings was further confirmed by the absence of publication bias. We may speculate that the increased risk of perforation, associated with septa, may be due to the difficulty in elevating thin sinus membrane off the sinus septa. Additionally, the operator may also have difficulty managing the curved curettes around the septa. Given the results of our meta-analysis, surgeons must thoroughly examine the 3D cone beam tomography scans for the presence of septa in the edentulous region, and to undertake the appropriate planning to reduce the risk of perforation.

Importantly, our meta-analysis reported high heterogeneity, with I^2^ values of 87%. Such high interstudy heterogeneity may be explained by several factors. Perforation rates can vary with the patient’s characteristics such as the history of smoking, thickness of the sinus membrane, pathological changes of membrane, residual ridge height, and maxillary sinus contours [8, 9]. These factors were not controlled by the studies that examined the risk of perforation with sinus septa. Additionally, no data were provided for a thorough subgroup or meta-regression analysis. Operator experience is another important confounder. The sinus membrane is a very delicate structure, and there is a learning curve associated with mastering the procedure. Since no information on operator experience was reported in the included studies, we were unable to examine the effect of this variable on our meta-analysis. Lastly, several studies used both rotary and piezosurgical instruments for creating the lateral window and did not segregate patients based on the technique used. Since perforation rates are lower with a piezosurgical instrument [37], it is plausible that this could be an important confounder contributing to the heterogeneity in the meta-analysis. Due to the lack of adjustment of such important covariates, current results should be interpreted with caution.

In the second part of the review, we examined the association between the thickness of the lateral wall and the risk of membrane perforation. During the sinus augmentation using the lateral window technique, a bony window is created with either a rotary or piezoelectric instrument to gain access to the sinus cavity. Research has shown that the use of piezoelectric instruments significantly reduces perforation rates compared to rotary handpieces [37]. However, with either instrument, the thickness of the wall itself could be a risk factor for perforations. Anatomical studies show that the mean thickness of the lateral wall is about 0.91± 0.43mm, and may be affected by factors like age, gender, location (premolar *vs* molar region), and the presence of teeth (thicker in dentate region) [15]. A thicker wall requires more bone drilling to create a bony window and could theoretically lead to an increased risk of membrane perforation. However, the evidence of such association is still unclear. Three studies by Nemati et al [24], Pizzini et al [16], and Basma et al [15] found a positive association between thick lateral walls and higher perforation rates, while studies by Marin et al [23] and Arx et al [22] found no such relationship. One reason for such discordant results could be the smaller sample size of the latter studies. Other factors that may influence outcomes include variation in the cut-offs of lateral wall thickness for statistical analysis, differences in the use of rotary and piezoelectric instruments, and surgical protocols. Therefore, our results cannot reliably confirm a strong association between lateral wall thickness and sinus membrane perforations. Further high-quality studies are needed to provide robust evidence.

This review has some limitations. Only a small number of studies were available, and most of them were retrospective and, therefore, prone to bias. There were methodological variations, especially in terms of drilling technique which could have affected reported perforation rates. A lack of separate data for rotary and piezoelectric groups did not allow us to perform subgroup analyses. Additionally, potential variability in the skills and experience of the operators may have influenced perforation rates. Patient-related factors such as sinus health, location of the edentulous region, and residual bone height are also important confounders that were not examined in the included studies. Lastly, a meta-analysis could be performed only for the presence of septa, while heterogeneous data for lateral wall thickness precluded a meta-analysis.

## Conclusions

This study examined the association between sinus anatomical features and the risk of membrane perforations and shows that maxillary sinuses with septa are associated with a significantly higher risk of perforations. However, the certainty of evidence was very low, and the evidence on the role of lateral wall thickness is conflicting and inconclusive. Surgeons should thoroughly evaluate each case of sinus lift for the presence of septa and thick lateral walls to reduce complication rates. Further studies are needed to add to the current evidence.

## Supporting information

S1 Table PRISMA checklist. (DOCX)

S2 Table Search strategy. (DOCX)

S3 Table Excluded studies with reasons. (DOCX)

S4 Table GRADE assessment of evidence. (DOCX)

S5 Table Genetic checklist. (DOCX)

## References

[1] Lozano-Carrascal N, Anglada-Bosqued A, Salomó-Coll O, Hernández-Alfaro F, Wang HL, Gargallo-Albiol J. Short implants (<8mm) versus longer implants (≥8mm) with lateral sinus floor augmentation in posterior atrophic maxilla: A meta-analysis of RCT`s in humans. Med Oral Patol Oral y Cir Bucal. 2020;25: e168–e179. doi: 10.4317/medoral.23248 32040465 PMC7103450

[2] Lee C-T, Choksi K, Shih M-C, Rosen PS, Ninneman S, Hsu Y-T. The Impact of Sinus Floor Elevation Techniques on Sinus Membrane Perforation: A Systematic Review and Network Meta-analysis. Int J Oral Maxillofac Implants. 2023;38: 681–696. doi: 10.11607/jomi.10048 37669518

[3] Romero-Millán J, Hernández-Alfaro F, Peñarrocha-Diago M, Soto-Peñaloza D, Peñarrocha-Oltra D, Peñarrocha-Diago M-A. Simultaneous and delayed direct sinus lift versus conventional implants: Retrospective study with 5-years minimum follow-up. Med Oral Patol Oral Cir Bucal. 2018;23: e752–e760. doi: 10.4317/medoral.22612 30341266 PMC6261006

[4] Del Fabbro M, Wallace SS, Testori T. Long-term implant survival in the grafted maxillary sinus: a systematic review. Int J Periodontics Restorative Dent. 2013;33: 773–83. doi: 10.11607/prd.1288 24116362

[5] Raghoebar GM, Onclin P, Boven GC, Vissink A, Meijer HJA. Long-term effectiveness of maxillary sinus floor augmentation: A systematic review and meta-analysis. J Clin Periodontol. 2019;46 Suppl 21: 307–318. doi: 10.1111/jcpe.13055 30624789

[6] Brandstaetter T, Ziv O, Sagy I, Segal N, Schneider S, Givol N, et al. Perforating dental implants and maxillary sinus pathology. Oral Maxillofac Surg. 2023 [cited 5 Jan 2024]. doi: 10.1007/s10006-023-01198-8 37985562

[7] Alshamrani AM, Mubarki M, Alsager AS, Alsharif HK, AlHumaidan SA, Al-Omar A. Maxillary Sinus Lift Procedures: An Overview of Current Techniques, Presurgical Evaluation, and Complications. Cureus. 2023;15: e49553. doi: 10.7759/cureus.49553 38156177 PMC10753870

[8] Valentini P. How to Prevent and Manage Postoperative Complications in Maxillary Sinus Augmentation Using the Lateral Approach: A Review. Int J Oral Maxillofac Implants. 2023;38: 1005–1013. doi: 10.11607/jomi.10145 37847842

[9] Ata-Ali J, Diago-Vilalta J-V, Melo M, Bagán L, Soldini M-C, Di-Nardo C, et al. What is the frequency of anatomical variations and pathological findings in maxillary sinuses among patients subjected to maxillofacial cone beam computed tomography? A systematic review. Med Oral Patol Oral Cir Bucal. 2017;22: e400–e409. doi: 10.4317/medoral.21456 28578369 PMC5549512

[10] Monje A, Diaz KT, Aranda L, Insua A, Garcia-Nogales A, Wang H-L. Schneiderian Membrane Thickness and Clinical Implications for Sinus Augmentation: A Systematic Review and Meta-Regression Analyses. J Periodontol. 2016;87: 888–99. doi: 10.1902/jop.2016.160041 27086614

[11] Fang Y, Bi Y, Mashrah MA, Su Y, Ge L, Dong Y, et al. Does the Presence of Pathological Change in the Schneiderian Membrane Increase the Risk of Membrane Perforation During Sinus Floor Elevation? A Systemic Review. J Oral Implantol. 2022;48: 147–157. doi: 10.1563/aaid-joi-D-20-00145 33270880

[12] Page MJ, McKenzie JE, Bossuyt PM, Boutron I, Hoffmann TC, Mulrow CD, et al. The PRISMA 2020 statement: An updated guideline for reporting systematic reviews. Int J Surg. 2021;88: 105906. doi: 10.1016/j.ijsu.2021.105906 33789826

[13] Wells G, Shea B, O’Connell D, Peterson J, Welch V, Losos M, et al. The Newcastle-Ottawa Scale (NOS) for assessing the quality of nonrandomised studies in meta-analyses. [cited 30 Oct 2020]. Available: http://www.ohri.ca/programs/clinical_epidemiology/oxford.asp

[14] Lu G, Li Q. The controlling nutritional status score as a predictor of survival in hematological malignancies: a systematic review and meta-analysis. Front Nutr. 2024;11: 1402328. doi: 10.3389/fnut.2024.1402328 38938670 PMC11208478

[15] Basma H, Saleh I, Abou-Arraj R, Li P, Benavides E, Wang H-L, et al. Association between lateral wall thickness and sinus membrane perforation during lateral sinus elevation: A retrospective study. Int J oral Implantol (Berlin, Ger. 2021;14: 77–85. Available: http://www.ncbi.nlm.nih.gov/pubmed/34006073 34006073

[16] Pizzini A, Basma HS, Li P, Geurs NC, Abou-Arraj R V. The impact of anatomic, patient and surgical factors on membrane perforation during lateral wall sinus floor elevation. Clin Oral Implants Res. 2021;32: 274–284. doi: 10.1111/clr.13698 33314302

[17] Prajapati S, Ninneman S, Zarrabi I, Daubert D, Wang I-C, Hsu Y-T. Risk factors and longitudinal regenerative outcomes of sinus membrane perforation during lateral window sinus floor elevation: A retrospective analysis up to 9 years. J Periodontol. 2023;94: 1045–1054. doi: 10.1002/JPER.22-0424 36748281

[18] Krennmair S, Gugenberger A, Weinländer M, Krennmair G, Malek M, Postl L. Prevalence, risk factors, and repair mechanism of different forms of sinus membrane perforations in lateral window sinus lift procedure: A retrospective cohort study. Clin Implant Dent Relat Res. 2021;23: 821–832. doi: 10.1111/cid.13016 34665489

[19] Tükel HC, Tatli U. Risk factors and clinical outcomes of sinus membrane perforation during lateral window sinus lifting: analysis of 120 patients. Int J Oral Maxillofac Surg. 2018;47: 1189–1194. doi: 10.1016/j.ijom.2018.03.027 29655818

[20] Schwarz L, Schiebel V, Hof M, Ulm C, Watzek G, Pommer B. Risk Factors of Membrane Perforation and Postoperative Complications in Sinus Floor Elevation Surgery: Review of 407 Augmentation Procedures. J Oral Maxillofac Surg. 2015;73: 1275–82. doi: 10.1016/j.joms.2015.01.039 25921824

[21] Shao Q, Li J, Pu R, Feng Y, Jiang Z, Yang G. Risk factors for sinus membrane perforation during lateral window maxillary sinus floor elevation surgery: A retrospective study. Clin Implant Dent Relat Res. 2021;23: 812–820. doi: 10.1111/cid.13052 34750940

[22] von Arx T, Fodich I, Bornstein MM, Jensen SS. Perforation of the sinus membrane during sinus floor elevation: a retrospective study of frequency and possible risk factors. Int J Oral Maxillofac Implants. 2014;29: 718–26. doi: 10.11607/jomi.3657 24818213

[23] Marin S, Kirnbauer B, Rugani P, Payer M, Jakse N. Potential risk factors for maxillary sinus membrane perforation and treatment outcome analysis. Clin Implant Dent Relat Res. 2019;21: 66–72. doi: 10.1111/cid.12699 30475442

[24] Nemati M, Khodaverdi N, Hosn Centenero SA, Tabrizi R. Which factors affect the risk of membrane perforation in lateral window maxillary sinus elevation? A prospective cohort study. J Craniomaxillofac Surg. 2023;51: 427–432. doi: 10.1016/j.jcms.2023.06.010 37423790

[25] Krennmair S, Malek M, Forstner T, Krennmair G, Weinländer M, Hunger S. Risk Factor Analysis Affecting Sinus Membrane Perforation During Lateral Window Maxillary Sinus Elevation Surgery. Int J Oral Maxillofac Implants. 2020;35: 789–798. doi: 10.11607/jomi.7916 32724933

[26] Bilge NH, Dagistanli S, Karasu YÖ, Orhan K. Comparison of Pathologic Changes in the Maxillary Sinus Before and After Dental Implant Surgery Using Cone Beam Computed Tomography. Int J Oral Maxillofac Implants. 2023;38: 1115–1122. doi: 10.11607/jomi.10321 38085742

[27] Park W-B, Herr Y, Chung J-H, Shin S-I, Han J-Y, Lim H-C. Long-term effects of sinus membrane perforation on dental implants placed with transcrestal sinus floor elevation: A case-control study. Clin Implant Dent Relat Res. 2021;23: 758–768. doi: 10.1111/cid.13038 34383373

[28] Ragucci GM, Elnayef B, Suárez-López Del Amo F, Wang H-L, Hernández-Alfaro F, Gargallo-Albiol J. Influence of exposing dental implants into the sinus cavity on survival and complications rate: a systematic review. Int J Implant Dent. 2019;5: 6. doi: 10.1186/s40729-019-0157-7 30719578 PMC6362182

[29] Viña-Almunia J, Peñarrocha-Diago M, Peñarrocha-Diago M. Influence of perforation of the sinus membrane on the survival rate of implants placed after direct sinus lift. Literature update. Medicina Oral, Patologia Oral y Cirugia Bucal. 2009. pp. 133–139. Available: http://www.medicinaoral.com/medoralfree01/v14i3/medoralv14i3p133.pdfhttp://www.medicinaoral.com/ 19242393

[30] Hernández-Alfaro F, Torradeflot MM, Marti C. Prevalence and management of Schneiderian membrane perforations during sinus-lift procedures. Clin Oral Implants Res. 2008;19: 91–8. doi: 10.1111/j.1600-0501.2007.01372.x 17961185

[31] Lee C-T, Choksi K, Shih M-C, Rosen PS, Ninneman S, Hsu Y-T. The Impact of Sinus Floor Elevation Techniques on Sinus Membrane Perforation: A Systematic Review and Network Meta-analysis. Int J Oral Maxillofac Implants. 2023;38: 681–696. doi: 10.11607/jomi.10048 37669518

[32] Nelke K, Diakowska D, Morawska-Kochman M, Janeczek M, Pasicka E, Łukaszewski M, et al. The CBCT Retrospective Study on Underwood Septa and Their Related Factors in Maxillary Sinuses-A Proposal of Classification. J Pers Med. 2023;13. doi: 10.3390/jpm13081258 37623508 PMC10455419

[33] Pommer B, Ulm C, Lorenzoni M, Palmer R, Watzek G, Zechner W. Prevalence, location and morphology of maxillary sinus septa: systematic review and meta-analysis. J Clin Periodontol. 2012;39: 769–73. doi: 10.1111/j.1600-051X.2012.01897.x 22624862

[34] Henriques I, Caramês J, Francisco H, Caramês G, Hernández-Alfaro F, Marques D. Prevalence of maxillary sinus septa: systematic review and meta-analysis. Int J Oral Maxillofac Surg. 2022;51: 823–831. doi: 10.1016/j.ijom.2021.10.008 34742634

[35] Wen S-C, Chan H-L, Wang H-L. Classification and management of antral septa for maxillary sinus augmentation. Int J Periodontics Restorative Dent. 2013;33: 509–17. doi: 10.11607/prd.1609 23820711

[36] Irinakis T, Dabuleanu V, Aldahlawi S. Complications During Maxillary Sinus Augmentation Associated with Interfering Septa: A New Classification of Septa. Open Dent J. 2017;11: 140–150. doi: 10.2174/1874210601711010140 28458730 PMC5388787

[37] Jordi C, Mukaddam K, Lambrecht JT, Kühl S. Membrane perforation rate in lateral maxillary sinus floor augmentation using conventional rotating instruments and piezoelectric device-a meta-analysis. Int J Implant Dent. 2018;4: 3. doi: 10.1186/s40729-017-0114-2 29376211 PMC5787532

