# Supplementary material for: Association between sinus septa and lateral wall thickness with risk of perforation during maxillary sinus lift surgery: A systematic review and meta-analysis
Source: PLoS One. 2024 Aug 8;19(8):e0308166. doi: 10.1371/journal.pone.0308166 (PMC11309397; doi:10.1371/journal.pone.0308166)
Supplement: S2 Table — (DOCX) [file pone.0308166.s002.docx]

Supplementary Table 2: Search queries

| Searching keywords | Details | Results |
| --- | --- | --- |
| (((sinus lift surgery) OR (sinus augmentation surgery)) AND (anatomy)) AND (complications) | ((("paranasal sinuses"[MeSH Terms] OR ("paranasal"[All Fields] AND "sinuses"[All Fields]) OR "paranasal sinuses"[All Fields] OR "sinus"[All Fields] OR "sinus s"[All Fields]) AND ("lifting"[MeSH Terms] OR "lifting"[All Fields] OR "lift"[All Fields]) AND ("surgery"[MeSH Subheading] OR "surgery"[All Fields] OR "surgical procedures, operative"[MeSH Terms] OR ("surgical"[All Fields] AND "procedures"[All Fields] AND "operative"[All Fields]) OR "operative surgical procedures"[All Fields] OR "general surgery"[MeSH Terms] OR ("general"[All Fields] AND "surgery"[All Fields]) OR "general surgery"[All Fields] OR "surgery s"[All Fields] OR "surgerys"[All Fields] OR "surgeries"[All Fields])) OR (("paranasal sinuses"[MeSH Terms] OR ("paranasal"[All Fields] AND "sinuses"[All Fields]) OR "paranasal sinuses"[All Fields] OR "sinus"[All Fields] OR "sinus s"[All Fields]) AND ("augment"[All Fields] OR "augmentation"[All Fields] OR "augmentations"[All Fields] OR "augmented"[All Fields] OR "augmenting"[All Fields] OR "augments"[All Fields]) AND ("surgery"[MeSH Subheading] OR "surgery"[All Fields] OR "surgical procedures, operative"[MeSH Terms] OR ("surgical"[All Fields] AND "procedures"[All Fields] AND "operative"[All Fields]) OR "operative surgical procedures"[All Fields] OR "general surgery"[MeSH Terms] OR ("general"[All Fields] AND "surgery"[All Fields]) OR "general surgery"[All Fields] OR "surgery s"[All Fields] OR "surgerys"[All Fields] OR "surgeries"[All Fields]))) AND ("anatomy and histology"[MeSH Subheading] OR ("anatomy"[All Fields] AND "histology"[All Fields]) OR "anatomy and histology"[All Fields] OR "anatomy"[All Fields] OR "anatomy"[MeSH Terms] OR "anatomies"[All Fields]) AND ("complicances"[All Fields] OR "complicate"[All Fields] OR "complicated"[All Fields] OR "complicates"[All Fields] OR "complicating"[All Fields] OR "complication"[All Fields] OR "complication s"[All Fields] OR "complications"[MeSH Subheading] OR "complications"[All Fields]) | 308 |
| (((sinus lift surgery) OR (sinus augmentation surgery)) AND (septa)) AND (perforation) | ((("paranasal sinuses"[MeSH Terms] OR ("paranasal"[All Fields] AND "sinuses"[All Fields]) OR "paranasal sinuses"[All Fields] OR "sinus"[All Fields] OR "sinus s"[All Fields]) AND ("lifting"[MeSH Terms] OR "lifting"[All Fields] OR "lift"[All Fields]) AND ("surgery"[MeSH Subheading] OR "surgery"[All Fields] OR "surgical procedures, operative"[MeSH Terms] OR ("surgical"[All Fields] AND "procedures"[All Fields] AND "operative"[All Fields]) OR "operative surgical procedures"[All Fields] OR "general surgery"[MeSH Terms] OR ("general"[All Fields] AND "surgery"[All Fields]) OR "general surgery"[All Fields] OR "surgery s"[All Fields] OR "surgerys"[All Fields] OR "surgeries"[All Fields])) OR (("paranasal sinuses"[MeSH Terms] OR ("paranasal"[All Fields] AND "sinuses"[All Fields]) OR "paranasal sinuses"[All Fields] OR "sinus"[All Fields] OR "sinus s"[All Fields]) AND ("augment"[All Fields] OR "augmentation"[All Fields] OR "augmentations"[All Fields] OR "augmented"[All Fields] OR "augmenting"[All Fields] OR "augments"[All Fields]) AND ("surgery"[MeSH Subheading] OR "surgery"[All Fields] OR "surgical procedures, operative"[MeSH Terms] OR ("surgical"[All Fields] AND "procedures"[All Fields] AND "operative"[All Fields]) OR "operative surgical procedures"[All Fields] OR "general surgery"[MeSH Terms] OR ("general"[All Fields] AND "surgery"[All Fields]) OR "general surgery"[All Fields] OR "surgery s"[All Fields] OR "surgerys"[All Fields] OR "surgeries"[All Fields]))) AND ("septa"[All Fields] OR "septae"[All Fields] OR "septas"[All Fields]) AND ("perforant"[All Fields] OR "perforants"[All Fields] OR "perforate"[All Fields] OR "perforated"[All Fields] OR "perforates"[All Fields] OR "perforating"[All Fields] OR "perforation"[All Fields] OR "perforations"[All Fields] OR "perforative"[All Fields] OR "perforator"[All Fields] OR "perforator s"[All Fields] OR "perforators"[All Fields]) | 39 |
| (((sinus lift surgery) OR (sinus augmentation surgery)) AND (lateral wall)) AND (perforation) | ((("paranasal sinuses"[MeSH Terms] OR ("paranasal"[All Fields] AND "sinuses"[All Fields]) OR "paranasal sinuses"[All Fields] OR "sinus"[All Fields] OR "sinus s"[All Fields]) AND ("lifting"[MeSH Terms] OR "lifting"[All Fields] OR "lift"[All Fields]) AND ("surgery"[MeSH Subheading] OR "surgery"[All Fields] OR "surgical procedures, operative"[MeSH Terms] OR ("surgical"[All Fields] AND "procedures"[All Fields] AND "operative"[All Fields]) OR "operative surgical procedures"[All Fields] OR "general surgery"[MeSH Terms] OR ("general"[All Fields] AND "surgery"[All Fields]) OR "general surgery"[All Fields] OR "surgery s"[All Fields] OR "surgerys"[All Fields] OR "surgeries"[All Fields])) OR (("paranasal sinuses"[MeSH Terms] OR ("paranasal"[All Fields] AND "sinuses"[All Fields]) OR "paranasal sinuses"[All Fields] OR "sinus"[All Fields] OR "sinus s"[All Fields]) AND ("augment"[All Fields] OR "augmentation"[All Fields] OR "augmentations"[All Fields] OR "augmented"[All Fields] OR "augmenting"[All Fields] OR "augments"[All Fields]) AND ("surgery"[MeSH Subheading] OR "surgery"[All Fields] OR "surgical procedures, operative"[MeSH Terms] OR ("surgical"[All Fields] AND "procedures"[All Fields] AND "operative"[All Fields]) OR "operative surgical procedures"[All Fields] OR "general surgery"[MeSH Terms] OR ("general"[All Fields] AND "surgery"[All Fields]) OR "general surgery"[All Fields] OR "surgery s"[All Fields] OR "surgerys"[All Fields] OR "surgeries"[All Fields]))) AND (("functional laterality"[MeSH Terms] OR ("functional"[All Fields] AND "laterality"[All Fields]) OR "functional laterality"[All Fields] OR "laterality"[All Fields] OR "lateral"[All Fields] OR "lateralisation"[All Fields] OR "lateralisations"[All Fields] OR "lateralise"[All Fields] OR "lateralised"[All Fields] OR "lateralises"[All Fields] OR "lateralising"[All Fields] OR "lateralities"[All Fields] OR "lateralization"[All Fields] OR "lateralizations"[All Fields] OR "lateralize"[All Fields] OR "lateralized"[All Fields] OR "lateralizes"[All Fields] OR "lateralizing"[All Fields] OR "laterally"[All Fields] OR "laterals"[All Fields]) AND "wall"[All Fields]) AND ("perforant"[All Fields] OR "perforants"[All Fields] OR "perforate"[All Fields] OR "perforated"[All Fields] OR "perforates"[All Fields] OR "perforating"[All Fields] OR "perforation"[All Fields] OR "perforations"[All Fields] OR "perforative"[All Fields] OR "perforator"[All Fields] OR "perforator s"[All Fields] OR "perforators"[All Fields]) | 41 |
| (((sinus membrane) OR (Schneiderian membrane)) AND (septa)) AND (perforation) | ((("paranasal sinuses"[MeSH Terms] OR ("paranasal"[All Fields] AND "sinuses"[All Fields]) OR "paranasal sinuses"[All Fields] OR "sinus"[All Fields] OR "sinus s"[All Fields]) AND ("membranal"[All Fields] OR "membrane s"[All Fields] OR "membraneous"[All Fields] OR "membranes"[MeSH Terms] OR "membranes"[All Fields] OR "membrane"[All Fields] OR "membranous"[All Fields])) OR ("nasal mucosa"[MeSH Terms] OR ("nasal"[All Fields] AND "mucosa"[All Fields]) OR "nasal mucosa"[All Fields] OR ("schneiderian"[All Fields] AND "membrane"[All Fields]) OR "schneiderian membrane"[All Fields])) AND ("septa"[All Fields] OR "septae"[All Fields] OR "septas"[All Fields]) AND ("perforant"[All Fields] OR "perforants"[All Fields] OR "perforate"[All Fields] OR "perforated"[All Fields] OR "perforates"[All Fields] OR "perforating"[All Fields] OR "perforation"[All Fields] OR "perforations"[All Fields] OR "perforative"[All Fields] OR "perforator"[All Fields] OR "perforator s"[All Fields] OR "perforators"[All Fields]) | 50 |
| (((sinus membrane) OR (Schneiderian membrane)) AND (lateral wall)) AND (perforation) | ((("paranasal sinuses"[MeSH Terms] OR ("paranasal"[All Fields] AND "sinuses"[All Fields]) OR "paranasal sinuses"[All Fields] OR "sinus"[All Fields] OR "sinus s"[All Fields]) AND ("membranal"[All Fields] OR "membrane s"[All Fields] OR "membraneous"[All Fields] OR "membranes"[MeSH Terms] OR "membranes"[All Fields] OR "membrane"[All Fields] OR "membranous"[All Fields])) OR ("nasal mucosa"[MeSH Terms] OR ("nasal"[All Fields] AND "mucosa"[All Fields]) OR "nasal mucosa"[All Fields] OR ("schneiderian"[All Fields] AND "membrane"[All Fields]) OR "schneiderian membrane"[All Fields])) AND (("functional laterality"[MeSH Terms] OR ("functional"[All Fields] AND "laterality"[All Fields]) OR "functional laterality"[All Fields] OR "laterality"[All Fields] OR "lateral"[All Fields] OR "lateralisation"[All Fields] OR "lateralisations"[All Fields] OR "lateralise"[All Fields] OR "lateralised"[All Fields] OR "lateralises"[All Fields] OR "lateralising"[All Fields] OR "lateralities"[All Fields] OR "lateralization"[All Fields] OR "lateralizations"[All Fields] OR "lateralize"[All Fields] OR "lateralized"[All Fields] OR "lateralizes"[All Fields] OR "lateralizing"[All Fields] OR "laterally"[All Fields] OR "laterals"[All Fields]) AND "wall"[All Fields]) AND ("perforant"[All Fields] OR "perforants"[All Fields] OR "perforate"[All Fields] OR "perforated"[All Fields] OR "perforates"[All Fields] OR "perforating"[All Fields] OR "perforation"[All Fields] OR "perforations"[All Fields] OR "perforative"[All Fields] OR "perforator"[All Fields] OR "perforator s"[All Fields] OR "perforators"[All Fields]) | 70 |

**EMBASE**

| No | Search | Results |
| --- | --- | --- |
| #1 | 'sinus lift surgery' OR (('sinus'/exp OR sinus)  AND ('lift'/exp OR lift) AND ('surgery'/exp OR  surgery)) | 1244 |
| #2 | sinus AND augmentation AND surgery | 2821 |
| #3 | sinus AND membrane | 7783 |
| #4 | schneiderian AND membrane | 411 |
| #5 | 'lateral wall thickness' | 94 |
| #6 | Septa | 11437 |
| #7 | Anatomy | 717100 |
| #8 | Complications | 1489594 |
| #9 | Perforation | 144155 |
| #10 | #1 OR #2 OR #3 OR #4 | 10308 |
| #11 | #5 OR #6 OR #7 | 727349 |
| #12 | #10 AND #11 | 721 |
| #13 | #9 AND #12 | 83 |
| #14 | #8 AND #12 | 128 |
| #15 | #1 AND #2 | 3443 |
| #16 | #9 OR #15 | 475 |

**Web of Science**

| **Query** | **Results** |
| --- | --- |
| (((sinus lift surgery) OR (sinus augmentation surgery)) AND (anatomy)) AND (complications) | 56 |
| (((sinus lift surgery) OR (sinus augmentation surgery)) AND (septa)) AND (perforation) | 16 |
| (((sinus lift surgery) OR (sinus augmentation surgery)) AND (lateral wall)) AND (perforation) | 18 |
| (((sinus membrane) OR (Schneiderian membrane)) AND (septa)) AND (perforation) | 10 |
| (((sinus membrane) OR (Schneiderian membrane)) AND (lateral wall)) AND (perforation) | 24 |

**ScienceDirect**

| **Query** | **Results** |
| --- | --- |
| (((sinus lift surgery) OR (sinus augmentation surgery)) AND (septa)) AND (perforation) | 375 |
| (((sinus lift surgery) OR (sinus augmentation surgery)) AND (lateral wall)) AND (perforation) | 2297 |
| (((sinus membrane) OR (Schneiderian membrane)) AND (septa)) AND (perforation) | 1091 |
| (((sinus lift surgery) OR (sinus augmentation surgery)) AND (anatomy)) AND (complications) | 5726 |

**CENTRAL**

| **Query** | **Results** |
| --- | --- |
| (((sinus lift surgery) OR (sinus augmentation surgery)) AND (anatomy)) AND (complications) | 4 |
| (((sinus lift surgery) OR (sinus augmentation surgery)) AND (septa)) AND (perforation) | 1 |
| (((sinus lift surgery) OR (sinus augmentation surgery)) AND (lateral wall)) AND (perforation) | 9 |
| (((sinus membrane) OR (Schneiderian membrane)) AND (septa)) AND (perforation) | 1 |
| (((sinus membrane) OR (Schneiderian membrane)) AND (lateral wall)) AND (perforation) | 16 |
