# Supplementary material for: Association between sinus septa and lateral wall thickness with risk of perforation during maxillary sinus lift surgery: A systematic review and meta-analysis
Source: PLoS One. 2024 Aug 8;19(8):e0308166. doi: 10.1371/journal.pone.0308166 (PMC11309397; doi:10.1371/journal.pone.0308166)
Supplement: S3 Table — (DOCX) [file pone.0308166.s003.docx]

Supplemental Table 3: Excluded studies with reasons

| Study | Reason |
| --- | --- |
| Yilmaz 2012[1] | Not on lateral wall thickness or septa |
| Taschieri 2014[2] | Not on lateral wall thickness or septa |
| Weitz 2014[3] | Not on lateral wall thickness or septa |
| Scarano 2015[4] | Not on lateral wall thickness or septa |
| Lin 2016[5] | Not on lateral wall thickness or septa |
| Toscano 2016[6] | Not on lateral wall thickness or septa |
| Sakkas 2016[7] | Not on lateral wall thickness or septa |
| Monje 2016[8] | Not on lateral wall thickness or septa |
| De Almeida 2017[9] | Not on lateral wall thickness or septa |
| Lum 2017[10] | Not on lateral wall thickness or septa |
| Oncu 2017[11] | Not on lateral wall thickness or septa |
| Ardekian 2006[12] | Not on lateral wall thickness or septa |
| Park 2019[13] | Not on lateral wall thickness or septa |
| Okada 2019[14] | Not on lateral wall thickness or septa |
| Jamcoski 2023[15] | Not on lateral wall thickness or septa |
| Ritter 2020[16] | Not on lateral wall thickness or septa |
| Krennmair 2020[17] | Duplicate data |
| Khalighi 2017[18] | Not reporting data for meta-analysis |
| Alhumaidan 2021[19] | Not reporting data for meta-analysis |
| Toscano 2010[20] | Not reporting data for meta-analysis |

References:

1. Yilmaz HG, Tözüm TF. Are gingival phenotype, residual ridge height, and membrane thickness critical for the perforation of maxillary sinus? J Periodontol. 2012;83: 420–5. doi:10.1902/jop.2011.110110

2. Taschieri S, Corbella S, Tsesis I, Del Fabbro M. Impact of the use of plasma rich in growth factors (PRGF) on the quality of life of patients treated with endodontic surgery when a perforation of sinus membrane occurred. A comparative study. Oral Maxillofac Surg. 2014;18: 43–52. doi:10.1007/s10006-012-0386-x

3. Weitz DS, Geminiani A, Papadimitriou DE V, Ercoli C, Caton JG. The incidence of membrane perforation during sinus floor elevation using sonic instruments: a series of 40 cases. Int J Periodontics Restorative Dent. 2014;34: 105–12. doi:10.11607/prd.1387

4. Scarano A, Mavriqi L, Bertelli I, Mortellaro C, Di Cerbo A. Occurrence of maxillary sinus membrane perforation following nasal suction technique and ultrasonic approach versus conventional technique with rotary instruments. J Craniofac Surg. 2015;26: 706–8. doi:10.1097/SCS.0000000000001755

5. Lin Y-H, Yang Y-C, Wen S-C, Wang H-L. The influence of sinus membrane thickness upon membrane perforation during lateral window sinus augmentation. Clin Oral Implants Res. 2016;27: 612–7. doi:10.1111/clr.12646

6. Toscano P, Toscano C, Del Fabbro M. Mini-invasive Implant Placement in Combination with Maxillary Sinus Membrane Perforation During Transcrestal Sinus Floor Elevation: A Retrospective Study. Int J Periodontics Restorative Dent. 2016;36: 199–211. doi:10.11607/prd.2280

7. Sakkas A, Konstantinidis I, Winter K, Schramm A, Wilde F. Effect of Schneiderian membrane perforation on sinus lift graft outcome using two different donor sites: a retrospective study of 105 maxillary sinus elevation procedures. GMS Interdiscip Plast Reconstr Surg DGPW. 2016;5: Doc11. doi:10.3205/iprs000090

8. Monje A, Monje-Gil F, Burgueño M, Gonzalez-Garcia R, Galindo-Moreno P, Wang H-L. Incidence of and Factors Associated with Sinus Membrane Perforation During Maxillary Sinus Augmentation Using the Reamer Drilling Approach: A Double-Center Case Series. Int J Periodontics Restorative Dent. 2016;36: 549–56. doi:10.11607/prd.2525

9. de Almeida Ferreira CE, Martinelli CB, Novaes AB, Pignaton TB, Guignone CC, Gonçalves de Almeida AL, et al. Effect of Maxillary Sinus Membrane Perforation on Implant Survival Rate: A Retrospective Study. Int J Oral Maxillofac Implants. 2017;32: 401–407. doi:10.11607/jomi.4419

10. Lum AG, Ogata Y, Pagni SE, Hur Y. Association Between Sinus Membrane Thickness and Membrane Perforation in Lateral Window Sinus Augmentation: A Retrospective Study. J Periodontol. 2017;88: 543–549. doi:10.1902/jop.2017.160694

11. Öncü E, Kaymaz E. Assessment of the effectiveness of platelet rich fibrin in the treatment of Schneiderian membrane perforation. Clin Implant Dent Relat Res. 2017;19: 1009–1014. doi:10.1111/cid.12528

12. Ardekian L, Oved-Peleg E, Mactei EE, Peled M. The clinical significance of sinus membrane perforation during augmentation of the maxillary sinus. J Oral Maxillofac Surg. 2006;64: 277–82. doi:10.1016/j.joms.2005.10.031

13. Park W-B, Han J-Y, Kang P, Momen-Heravi F. The clinical and radiographic outcomes of Schneiderian membrane perforation without repair in sinus elevation surgery. Clin Implant Dent Relat Res. 2019;21: 931–937. doi:10.1111/cid.12752

14. Okada T, Kawana H. Two-Step Procedure for the Treatment of a Maxillary Sinus with Complex Sinus Septa: A Highly Predictive Method for Sinus Floor Augmentation After Perforation of the Maxillary Sinus Membrane. Int J Periodontics Restorative Dent. 2019;39: e175–e180. doi:10.11607/prd.3888

15. Jamcoski VH, Faot F, Marcello-Machado RM, Melo ACM, Fontão FNGK. 15-Year Retrospective Study on the Success Rate of Maxillary Sinus Augmentation and Implants: Influence of Bone Substitute Type, Presurgical Bone Height, and Membrane Perforation during Sinus Lift. Biomed Res Int. 2023;2023: 9144661. doi:10.1155/2023/9144661

16. Ritter A, Rozendorn N, Avishai G, Rosenfeld E, Koren I, Soudry E. Preoperative Maxillary Sinus Imaging and the Outcome of Sinus Floor Augmentation and Dental Implants in Asymptomatic Patients. Ann Otol Rhinol Laryngol. 2020;129: 209–215. doi:10.1177/0003489419883292

17. Krennmair S, Malek M, Forstner T, Krennmair G, Weinländer M, Hunger S. Risk Factor Analysis Affecting Sinus Membrane Perforation During Lateral Window Maxillary Sinus Elevation Surgery. Int J Oral Maxillofac Implants. 2020;35: 789–798. doi:10.11607/jomi.7916

18. Khalighi Sigaroudi A, Dalili Kajan Z, Rastgar S, Neshandar Asli H. Frequency of different maxillary sinus septal patterns found on cone-beam computed tomography and predicting the associated risk of sinus membrane perforation during sinus lifting. Imaging Sci Dent. 2017;47: 261–267. doi:10.5624/isd.2017.47.4.261

19. Alhumaidan G, Eltahir MA, Shaikh SS. Retrospective analysis of maxillary sinus septa - A cone beam computed tomography study. Saudi Dent J. 2021;33: 467–473. doi:10.1016/j.sdentj.2020.11.001

20. Toscano NJ, Holtzclaw D, Rosen PS. The effect of piezoelectric use on open sinus lift perforation: a retrospective evaluation of 56 consecutively treated cases from private practices. J Periodontol. 2010;81: 167–71. doi:10.1902/jop.2009.090190
