# Supplementary material for: Association between sinus septa and lateral wall thickness with risk of perforation during maxillary sinus lift surgery: A systematic review and meta-analysis
Source: PLoS One. 2024 Aug 8;19(8):e0308166. doi: 10.1371/journal.pone.0308166 (PMC11309397; doi:10.1371/journal.pone.0308166)
Supplement: S4 Table — (DOCX) [file pone.0308166.s004.docx]

Supplementary Table 4: GRADE assessment of evidence

| **Certainty assessment** | | | | | | | **No of patients** | | **Effect** | | **Certainty** |
| --- | --- | --- | --- | --- | --- | --- | --- | --- | --- | --- | --- |
| **No of studies** | **Study design** | **Risk of bias** | **Inconsistency** | **Indirectness** | **Imprecision** | **Other considerations** | **Septa** | **No Septa** | **Relative (95% CI)** | **Absolute (95% CI)** |  |
| 9 | non-randomized studies | serious^a^ | not serious | not serious | not serious | none | 169/425 (39.8%) | 184/1492 (12.3%) | OR 4.03 (1.77 to 9.19) | 238 more per 1,000 (from 76 more to 441 more) | ⨁◯◯◯ Very low |

CI, confidence interval; OR, odds ratio

^a^All studies received an NOS score of 7 and had a bias in the comparability of groups
